# Aberrant Expression of NF-κB in Liver Fluke Associated Cholangiocarcinoma: Implications for Targeted Therapy

**DOI:** 10.1371/journal.pone.0106056

**Published:** 2014-08-29

**Authors:** Wunchana Seubwai, Chaisiri Wongkham, Anucha Puapairoj, Narong Khuntikeo, Ake Pugkhem, Chariya Hahnvajanawong, Jariya Chaiyagool, Kazuo Umezawa, Seiji Okada, Sopit Wongkham

**Affiliations:** 1 Department of Biochemistry, Faculty of Medicine, Khon Kaen University, Khon Kaen, Thailand; 2 Department of Forensic Medicine, Faculty of Medicine, Khon Kaen University, Khon Kaen, Thailand; 3 Department of Pathology, Faculty of Medicine, Khon Kaen University, Khon Kaen, Thailand; 4 Department of Surgery, Faculty of Medicine, Khon Kaen University, Khon Kaen, Thailand; 5 Department of Microbiology, Faculty of Medicine, Khon Kaen University, Khon Kaen, Thailand; 6 Liver Fluke and Cholangiocarcinoma Research Center, Faculty of Medicine, Khon Kaen University, Khon Kaen, Thailand; 7 Department of Molecular Target Medicine Screening, Aichi Medical University, Nagakute, Japan; 8 Division of Hematopoiesis, Center for AIDS Research, Kumamoto University, Honjo, Kumamoto, Japan; Nihon University School of Medicine, Japan

## Abstract

**Background:**

Up-regulation and association of nuclear factor kappa B (NF-κB) with carcinogenesis and tumor progression has been reported in several malignancies. In the current study, expression of NF-κB in cholangiocarcinoma (CCA) patient tissues and its clinical significance were determined. The possibility of using NF-κB as the therapeutic target of CCA was demonstrated.

**Methodology:**

Expression of NF-κB in CCA patient tissues was determined using immunohistochemistry. Dehydroxymethylepoxyquinomicin (DHMEQ), a specific NF-κB inhibitor, was used to inhibit NF-κB action. Cell growth was determined using an MTT assay, and cell apoptosis was shown by DNA fragmentation, flow cytometry and immunocytofluorescent staining. Effects of DHMEQ on growth and apoptosis were demonstrated in CCA cell lines and CCA-inoculated mice. DHMEQ-induced apoptosis in patient tissues using a histoculture drug response assay was quantified by TUNEL assay.

**Principal Findings:**

Normal bile duct epithelia rarely expressed NF-κB (subunits p50, p52 and p65), whereas all CCA patient tissues (n  =  48) over-expressed all NF-κB subunits. Inhibiting NF-κB action by DHMEQ significantly inhibited growth of human CCA cell lines in a dose- and time-dependent manner. DHMEQ increased cell apoptosis by decreasing the anti-apoptotic protein expressions–Bcl-2, XIAP–and activating caspase pathway. DHMEQ effectively reduced tumor size in CCA-inoculated mice and induced cell apoptosis in primary histocultures of CCA patient tissues.

**Conclusions:**

NF-κB was over-expressed in CCA tissues. Inhibition of NF-κB action significantly reduced cell growth and enhanced cell apoptosis. This study highlights NF-κB as a molecular target for CCA therapy.

## Introduction

*O. viverrini*, a carcinogenic liver fluke, is endemic mainly in Thailand, the Laotian People's Democratic Republic, Cambodia and central Vietnam [1]. The animal and epidemiological studies strongly showed the association of *O. viverrini* infection and cholangiocarcinoma (CCA) development in this geographical area (reviewed in [2]) with individuals being infected by consuming raw or uncooked fish contaminated with the metacercariae, the infective stage of the parasite. At least 10 million people were estimated to be infected with *O. viverrini* in Thailand and the Laotian PDR with an unknown number of additional cases in Cambodia and Vietnam [3] and aproximately 5% of these were predicted to develop CCA.

CCA is rare worldwide, comprising around 10–15% of primary liver cancers in most parts of the world. The incidence, however, is very high in Southeast Asia, especially in the northeast of Thailand where the prevalence of *O. viverrini* infection and CCA are the highest in the country [4]. The mortality rates of primary liver cancer in both males and females in this region in 2010 were 34.86/100,000 or 14,410 cases per year [5]. In Khon Kaen province, the endemic area of *O. viverrini*, the mortality rates of CCA in the area were as high as 62.0/100,000 in males and 25.6/100,000 in females [6]. CCA is an aggressive and lethal cancer. As it is difficult to diagnose at an early stage, almost all patients are diagnosed at a late presentation with advanced and incurable disease. In addition, the recurrence rate is high even in patients who have undergone complete surgical resection. These factors lead to the high mortality rate of CCA patients. At present, there is no effective treatment for CCA, hence, an improvement of new therapeutic regimens using specifically targeted molecules is deemed essential to improve the clinical outcome.

In recent years, nuclear factor-κB (NF-κB) has emerged as a potential molecular possibility for treatment of several malignancies [7]–[9]. Active NF-κB complexes are dimers of various combinations of the Rel family of polypeptides consisting of p50 (NF-κB1), p52 (NF-κB2), c-Rel, v-Rel, Rel A (p65) and Rel B [10]. NF-κB is activated by a wide variety of stimuli and cytokines, including UV radiation, chemical carcinogens, tumor necrosis factor-α, chemotherapeutic agents [11], [12] and radiation therapy [13], [14], which cause dissociation of the binding of inhibitory IκB proteins and consequently leads to the relocation of the NF-κB complex into the nucleus. Activated NF-κB promotes over 150 target transcripts, which include various genes involved in cell proliferation [15], angiogenesis [16], metastasis [17], suppression of apoptosis [18] and resistance to chemotherapeutic drugs [19], [20]. These data indicate NF-κB as a valid therapeutic molecule for cancer treatment.

In the current study, aberrant expression of NF-κB in CCA patient tissues was first reported. It was further shown that inhibiting the action of NF-κB using DHMEQ (dehydroxymethylepoxyquinomicin), a novel NF-κB inhibitor, could effectively suppress growth of CCA cell lines and CCA in inoculated mice. The treatment also induced apoptosis of CCA in cell lines and CCA patient tissues. The molecular basis by which DHMEQ affected expression of apoptosis related proteins and nuclear translocation of NF-κB were verified. These findings provide the first evidence indicating NF-κB as an attractive molecule for CCA therapy.

## Materials and Methods

### Tissues and cell lines

Paraffin-embedded liver tissues from patients with histologically proven CCA (n  =  48) who underwent liver resection were obtained from the specimen bank of the Liver Fluke and Cholangiocarcinoma Research Center, Faculty of Medicine, Khon Kaen University, Thailand.

Human CCA cell lines derived from different histological types of primary CCA tumor [21] –namely KKU-M139 (adeno-squamous CCA), KKU-M156 (moderately differentiated CCA), KKU-M213 (mixed papillary and non-papillary CCA), KKU-M214 (well-differentiated CCA) and KKU-100 (poorly differentiated CCA) were established as described by B Sripa [22]. CCA cell lines were cultured in Ham-F12 media supplemented with 10% fetal calf serum, 1% L-glutamine, and 100 U/mL penicillin and 100 µg/mL streptomycin at 37 °C and 5% CO_2_.

Written informed consent was obtained from each subject and the protocol has been reviewed and approved by Ethics Committee for Human Research of Khon Kaen University (HE43210) based on the Declaration of Helsinki and ICH-Good Clinical Practice Guidelines.

### Immunohistochemistry

NF-κB subunits (p50, p52 and p65) were detected in formalin-fixed paraffin-embedded tissue sections according to standard immunohistochemistry techniques. The tissue sections were reacted at room temperature overnight with 1∶200 of primary antibodies: anti-p50 (NLS), anti-p52 (C-5) and anti-p65 (F-6) (Santa Cruz Biotecnology, Santa Cruz, CA, USA) and with 1∶2000 peroxidase at room temperature for 1 h. The peroxidase activity was observed using diaminobenzidine tetrahydroxychloride solution (DAB; Dako, Glostrup, Denmark) as the substrate. The sections were counterstained with hematoxylin. The frequency of NF-κB positive cells was semi-quantitatively scored on the basis of the percentage of positive cells as 0%  =  negative; 1–25%  =  +1; 26–50%  =  +2; and >50%  =  +3. The intensity of NF-κB expression was scored as weak  =  1, moderate  =  2 and strong  =  3. The immunohistochemistry index of NF-κB expression of each section was calculated as intensity multiplied by frequency and categorized as low (< 6) or high (> 6).

### Western Blot Analysis

Whole cell, cytoplasmic or nuclear lysates of CCA cells were prepared as described previously [21]. Whole cell lysate was prepared in NP-40 lysis buffer (150 mM NaCl, 1% NP-40, 50 mM Tris-HCl, pH 8.0). For cytoplasmic and nuclear lysates, cells were lyzed in hypotonic buffer (10 mM HEPES-KOH, pH 7.9, 1.5 mM MgCl_2_, 10 mM KCl, 0.1% NP-40, 0.5 mM DTT and 0.5 mM PMSF) and incubated on ice for 15 min. Cytoplasmic fraction was collected by centrifugation at 5,000 rpm for 1 min. The pellet was used as nuclear fraction and lysed with nuclear lysis buffer (50 mM HEPES-KOH pH 7.9, 10% glycerol, 420 mM KCl, 5 mM MgCl_2_, 0.1 mM DTT, 0.5 mM PMSF and 2 µg/ml Aprotinin), incubated on ice for 30 min. Nuclear lysate was obtained by centrifugation at 14,000 rpm for 10 min.

Samples (10 µg protein) were electrophoresed on a 10% sodium dodecyl sulfate polyacrylamide gel. The separated proteins were blotted onto a polyvinylidine fluoride membrane (GE Healthcare, Tokyo, Japan) and were detected with a specific primary antibody using the enhanced chemiluminescence (ECL) Western Blotting Detection System (GE Healthcare Bio-Science, Buckinghamshire, United Kingdom). The primary antibodies used were: anti-p65 (F-6), anti-p52 (C-5), anti-p50 (NLS), anti-IκBα (C-21), anti-IKKαβ (H-470), anti-BAX (P-19), anti-Bcl-2 (C-21), anti-XIAP (H-202), anti-beta actin (C-5) and anti-Histone H1 (N-16) (Santa Cruz Biotechnology, Santa Cruz, CA, USA), caspase 3 (9662) and caspase 9 (9502) (Cell signaling Technology, Danvers, MA, USA). Quantification of the Western blots was performed using GelPro 32 (Media Cybernetics, Bethesda, Md). Relative density was evaluated and normalized with β-actin or Histone H1.

### Cell viability test

The MTT assay was applied to test cell viability. In brief, 3 × 10^3^ cells per well were seeded in a 96-well plate and incubated with various concentrations of DHMEQ (2.5–20 µg/mL) for 24, 48, and 72 h, at 37 °C and 5% CO_2_. Cells treated with vehicle were used as control. Subsequently, 10 µL MTT (Sigma-Aldrich, St. Louis, Mo, USA) was added to yield the final concentration of 0.5 mg/mL. After 4 hours of additional incubation, 100 µL of 0.01 N HCl in isopropanol was added to dissolve the crystals; absorption at 590 nm was determined with an automatic ELISA plate reader (Multiskan; Thermo Electron, Vantaa, Finland).

### DNA fragmentation assay

Approximately 10^6^ cells were lyzed in 100 µL of 10 mM Tris–HCl buffer (pH 7.4) containing 10 mM EDTA and 0.5% Triton X-100. The DNA fragmentation assay was performed according to the method described previously [21]. Finally, the DNA pellets were dissolved in 20 µL of TE buffer (10 mM Tris–HCl and 1 mM EDTA) and loaded onto a 1.5% agarose gel in 1% TBE buffer (89 mM Tris Base, 89 mM boric acid, and 2 mM EDTA) and electrophoresed at 100 volt for 30 min and stained with ethidium bromide.

### Cell cycle and apoptosis analysis

Numbers of cells in each phase of cell cycle and apoptotic cells were quantified using propidium iodide staining. CCA cells of 5×10^4^ cells per well were seeded in a 6-well plate and treated with either DHMEQ or vehicle (0.05% dimethyl sulfoxide, DMSO) as a control for 3 days. Cells were scraped and washed once with phosphate-buffered saline (PBS), and fixed with an ice-cold 70% ethanol overnight at 4°C. After centrifugation, cell pellets were resuspended in 1 mL of 1 µg/mL propidium iodide and incubated at room temperature for 1 h. DNA content was determined in an LSR II flow cytometer (BD Bioscience, San Jose, CA) and analyzed using FlowJo software (Tree Star, San Carlos, CA).

### Cell death assay using the IN Cell Analyzer 2000

Cytotoxicity of DHMEQ on CCA cells was examined in the IN Cell Analyzer 2000. Cells were seeded in a 24-well cell-culture plate at a density of 1 × 10^4^ cells/well and allowed to adhere for 24 h. Cells were subsequently treated with 10 or 20 µg/mL DHMEQ for 48 h at 37°C, 5% CO_2_. After treatment, cells were stained with propidium iodide and Annexin V (Molecular Probes, Eugene, OR) for 30 min before image acquisition in an IN Cell Analyzer 2000 (GE Healthcare, UK). The 20× objective (NA 0.45) was used to collect images for all fluorescent channels. Five fields-of-view per well were monitored. Image analysis for the multiplex assay was performed using the IN Cell Analyzer 1000 Workstation ver.3.7 (GE Healthcare, UK).

### *In vivo* assay

NOD/Scid/Jak3 deficient (NOJ) male mice (8 to 10-week-old) were housed and monitored in the animal research facility according to the institutional guidelines. All experimental protocols were approved by the Institutional Animal Care and Use Committee, Kumamoto University. Mice were subcutaneously injected with 4 × 10^6^ CCA cells at both flank sides. A day after tumor injection, mice were intraperitoneally injected with PBS or DHMEQ (10 mg/kg body weight) daily for 17 days. The body weight, feeding behavior, and motor activity of each animal were monitored as indicators of general health. Tumors were removed and weighed 18 days after inoculation.

### Histoculture drug response assay

The Histoculture drug response assay (HDRA) was performed according to the method described previously [21]. The primary tissue culture was prepared for a drug response assay in a 24-well microplate. Fresh cancerous tissues from CCA patients (n  =  4) were dissected into approximately 10-mg slices and placed on a collagen sponge gel (Sumitomo Pharma, Osaka, Japan), which was submerged in RPMI-1640 medium containing 20% fetal calf serum in the presence or absence of 5, 10 and 20 µg/mL DHMEQ. The tissues were cultured for 4 days at 37°C, 5% CO_2_, and processed for immunohistochemical staining using the DeadEnd Colorimetric TUNEL System (Promega, Madison, WI). TUNEL-positive cells were randomly quantified under a microscope in at least four high-power fields (x40). The apoptotic index was defined as the percentage of dead cells in the treated group divided by the percentage of dead cells in control group.

### Statistical analysis

The results are presented as a mean ± SD for at least three separate experiments. Statistical significance was determined using the Student's t-test and *P* < 0.05 was required for statistical significance.

## Results

### NF-κB was over-expressed in CCA tissues and cells line

**Figure 1:**
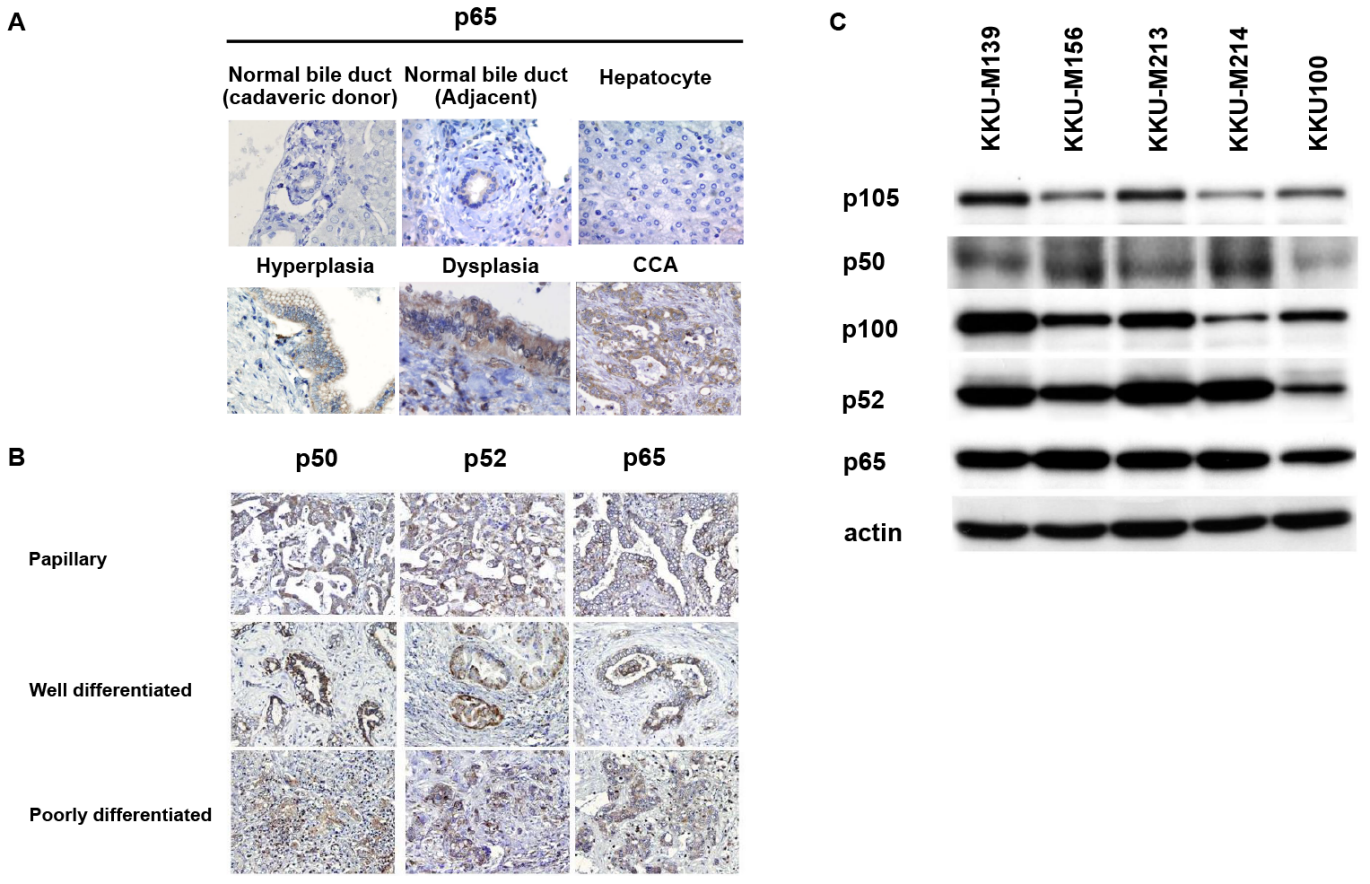
Expression of NF-κB in CCA patients and cell lines. The expressions of NF-κB in CCA tissues were determined by immunohistochemistry. A) Hepatocytes, as well as bile duct epithelia of normal liver tissues from cadaveric donor and of non-tumorous liver tissue from CCA patients showed weak immunoreactivity of NF-κB (p65). The expression of NF-κB, however, was gradually increased in hyperplasia, dysplasia and CCA. B) All NF-κB subunits, p50, p52 and p65, were overexpressed in different histological types of CCA tissues. Original magnification 20X, except normal bile duct, hepatocyte and dysplasia, 40X. C) Expression levels of NF-κB subunits, p50, p52 and p65, were determined in five CCA cell lines by western blotting. The precursors and active forms of NF-κB subunits, p105/p50, p100/p52 and p65, were strongly expressed in all CCA cell lines tested. Actin was used as an internal control.

The expression of NF-κB subunits p50, p52 and p65 in liver tissue sections (n  = 48) from histologically proven CCA patients by immunohistochemistry were first examined. The clinical findings of the patients are shown in Table 1. Normal bile duct epithelia found in non-tumorous tissues and hepatocytes from CCA patients were negative for immuno-staining of all NF-κB subunits. To ensure the negative expression of NF-κB in the normal bile duct epithelia, the liver sections from cadaveric donors (n  =  5) were also included. Fig. 1A shows the immuno-stainings of p65 as a representative of NF-κB subunit expression in liver tissues from cadaveric donors and CCA patients. Normal bile duct epithelia of CCA patients and cadaveric donors did not express any NF-κB subunits. In contrast, all NF-κB subunits were frequently and strongly expressed in the cytoplasm of hyperplastic/dysplastic and CCA bile duct epithelia (Fig. 1B). Approximately 70% of CCA tissues highly expressed p50 and p52 NF-κB subunits and all patient tissues expressed high p65. Even though all CCA patients had positive NF-κB signals, no relationships between NF-κB expressions and clinico-pathological findings eg. age, sex, tumor staging, tumor size, tumor type and metastasis status, and the cumulative survival of the patients were noted (data not shown).

**Table 1:** Characteristics of CCA Patients (n  =  48).

Variables		Numbers
Age	< 56	19
	> 56	29
Sex	Men	35
	Women	13
Histopathology	Papillary	3
	Non-Papillary	45
Tumor type	Central	2
	Peripheral	37
	Combined	9
Tumor stage	I–III	6
	IVA	13
	IVB	29

The expression levels of NF-κB, p50, p52 and p65 in total cell lysate and the nuclear fraction of human CCA cell lines were further analyzed by western blotting using β-actin and histone H1 as the internal controls for total cell lysate and nuclear fraction, respectively. Five CCA cell lines established from different histological types of primary tumors, namely, KKU-M139, KKU-M156, KKU-M213, KKU-M214 and KKU-100 showed positive expression of p50, p52 and p65 NF-κB subunits (Fig. 1C).

### Inhibition of NF-κB with low concentrations of DHMEQ suppressed growth and induced G2/M arrest of CCA cell lines

**Figure 2:**
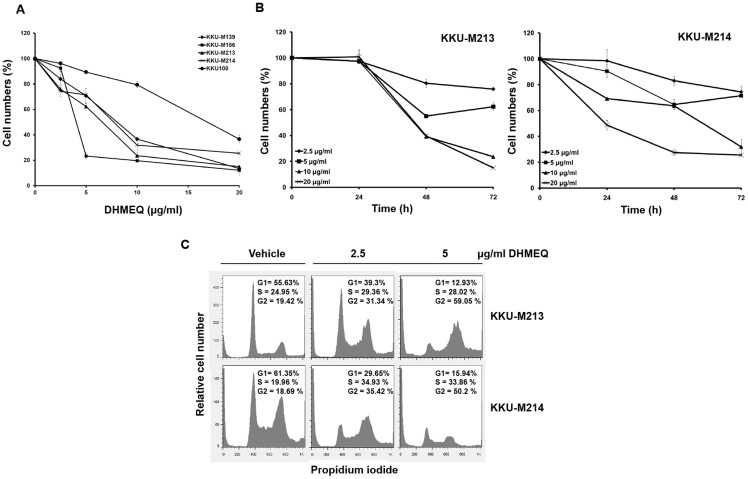
High concentration of DHMEQ caused cell death and low concentration induced G2/M arrest in CCA cell lines. A) DHMEQ could inhibit growth of 5 CCA cell lines as monitored by MTT assay at 72 h. B) DHMEQ suppressed growth of CCA cell lines in a dose and time dependent fashions. C) Growth arrest at G2/M phase was observed in CCA cell lines treated with low concentrations of DHMEQ (2.5 and 5 µg/mL).

The aberrant expression of NF-κB in CCA implicated the important role of NF-κB in CCA. The role of NF-κB on growth of CCA cell lines was next examined. DHMEQ, a well-known NF-κB inhibitor, was used to inhibit NF-κB action and cell growth was determined by an MTT assay. Five CCA cell lines were incubated with DHMEQ at 2.5, 5, 10 and 20 µg/mL for 24, 48 and 72 h. Cells grown in vehicle without DHMEQ were used as controls. As shown in Fig. 2A, DHMEQ significantly inhibited growth of all CCA cell lines in a dose dependent fashion. DHMEQ at 10µg/mL effectively suppressed growth of all cell lines to < 40% of the controls except KKU-100. Apoptotic cells were obviously observed in the DHMEQ treated cells at 10 µg/mL. DHMEQ also significantly inhibited the growth of CCA cell lines in a time dependent manner as shown for KKU-M213 and KKU-M214 (*P* < 0.001) (Fig. 2B).

The association of NF-κB with the cell cycle was investigated by flow cytometry with propidium iodide staining. Treatment of KKU-M213 and KKU-M214 cells with low concentrations of DHMEQ at 2.5 and 5 µg/mL caused an accumulation of cells at G2/M phase compared with the controls treated with vehicle alone (Fig. 2C).

### Inhibition of NF-κB with high concentrations of DHMEQ induced apoptosis in CCA cell lines

**Figure 3:**
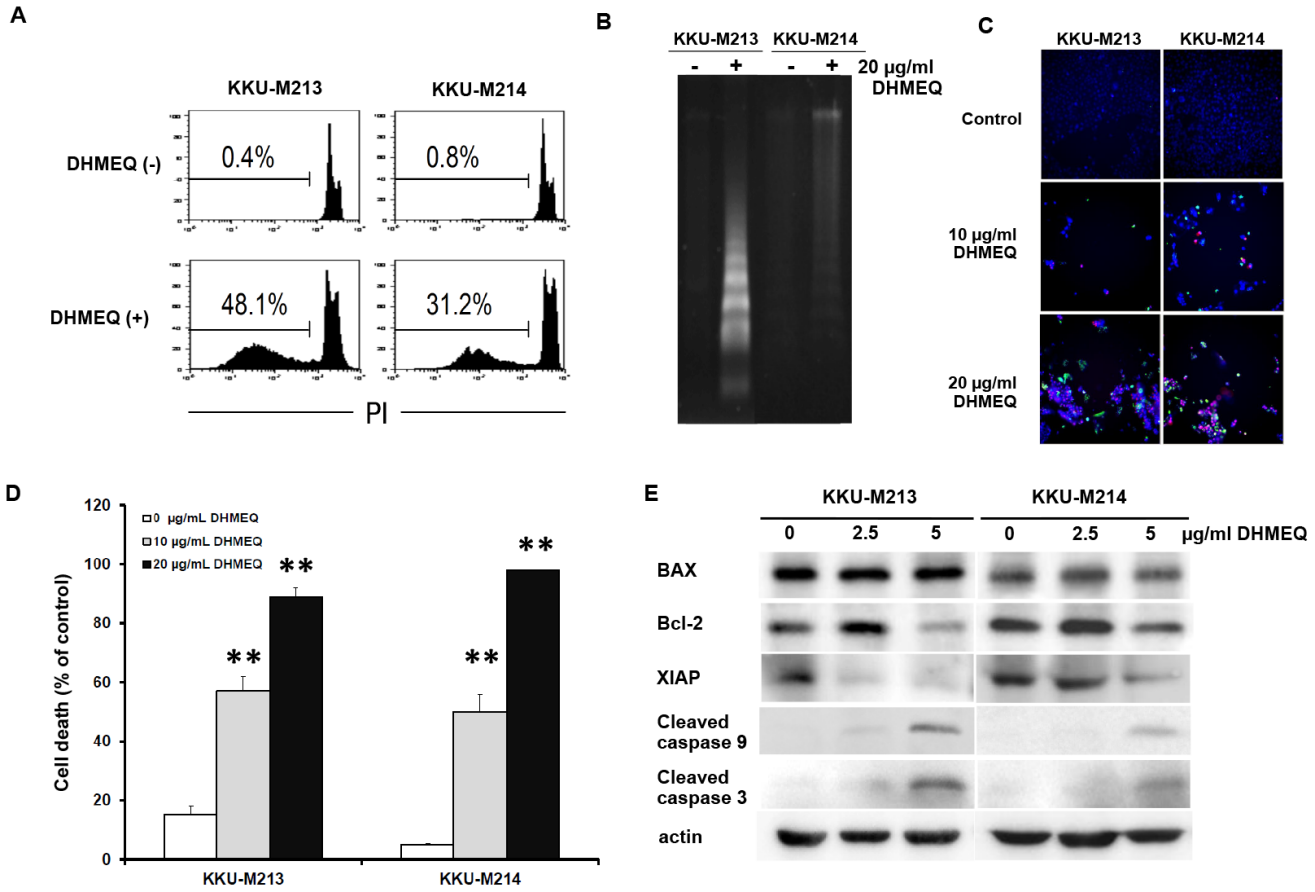
Inhibition of NF-κB action by high concentration of DHMEQ induced apoptosis in CCA cell lines. An increase of apoptotic cells was found in CCA cell lines, KKU-M213 and KKU-M214, treated with high concentrations of DHMEQ (10 and 20 µg/mL) for 48 h. Apoptotic cells were determined using various methods. A) flow cytometry with PI staining; B) DNA fragmentation; C) Annexin V staining with IN Cell Analyzer (magnification ×20, blue  =  Hoechst 33342, green  =  Annexin V and Red  =  PI staining); D) quantitative analysis of apoptotic cells from C); E) immunoblotting of apoptotic proteins and caspases of CCA cells treated with DHMEQ. **P < 0.01; independent-sample t-test compared to the control group.

To investigate the role of NF-κB on anti-apoptosis, KKU-M213 and KKU-M214 were incubated with 10 µg/mL DHMEQ for 48 h. Apoptotic cells were examined using flow cytometry with propidium iodide staining and the DNA fragmentation assay. DHMEQ at 10 µg/mL significantly increased sub-G1 cells to 48% of untreated KKU-M213 and 31% of untreated KKU-M214 (Fig. 3A). DHMEQ-induced cell apoptosis was confirmed using a DNA fragmentation assay. CCA cells treated with 20 µg/mL of DHMEQ for 48 h showed a significant increase of DNA fragmentation compared to the controls (Fig. 3B). A similar finding was observed when cells were analyzed using the IN Cell Analyzer (Fig. 3C). DHMEQ significantly increased cell death (Annexin V and/or PI positive cells) of CCA cell lines (*P* < 0.001). The percentages of dead cells were increased to 89 ± 4.5% and 97.9±0.3% after KKU-M213 and KKU-M214 were incubated with 20 µg/mL DHMEQ for 48 h (Fig. 3D).

The expression levels of apoptosis related genes in CCA cells treated with DHMEQ (2.5 and 5 µg/ml) comparing to those treated with vehicle were further examined. Expressions of apoptotic associated proteins, BAX, Bcl-2, XIAP, as well as caspase-3 and caspase-9, were determined using Western blot analysis. DHMEQ significantly suppressed expressions of anti-apoptotic proteins–Bcl-2 and XIAP–but had no effect on BAX, a pro-apoptotic protein (Fig. 3E). In addition, activated forms of caspase-3 and caspase-9 were progressively increased in DHMEQ treated CCA cell lines.

### Translocation of NF-κB was inactivated by DHMEQ

**Figure 4:**
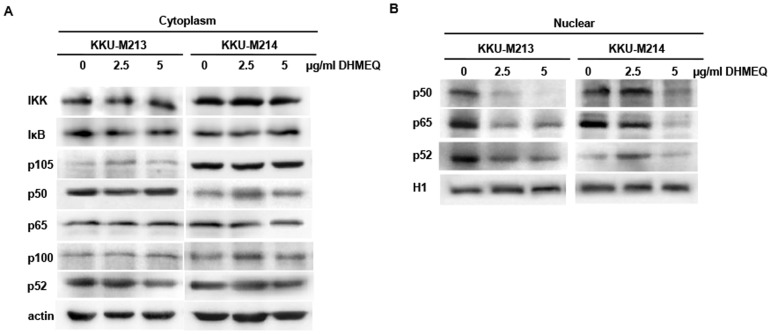
DHMEQ inhibited nuclear translocation of NF-κB in CCA cell lines. Western blot of NF-κB of KKU-M213 and KKU-M214 treated with 2.5 and 5 µg/mL of DHMEQ for 48 h. Protein (10 µg) of A) cytoplasmic fraction and B) nuclear fraction was loaded. Actin and histone H1 were used as internal controls.

To investigate whether DHMEQ affected growth and apoptosis of CCA cells via inactivation of NF-κB, the expression of NF-κB subunits in cytoplasmic and nuclear fractions were determined using Western blot analysis. NF-κB regulatory proteins and NF-κB subunits of CCA cells treated with 2.5 and 5 µg/ml of DHMEQ for 48 h were compared to controls. As shown in Fig. 4A, expressions of cytoplasmic IKK, IκB, p50, p65 and p52 and their precursors, p105 and p100, of KKU-M213 cells treated with DHMEQ were not different from those of the controls. On the other hand, the treatment decreased the levels of all NF-κB subunits in the nuclear fraction in a dose-dependent manner. Similar results were obtained for KKU-M214 treated with DHMEQ (Fig. 4B).

### Inhibition of NF-κB action suppressed tumor growth in CCA-inoculated mice

**Figure 5:**
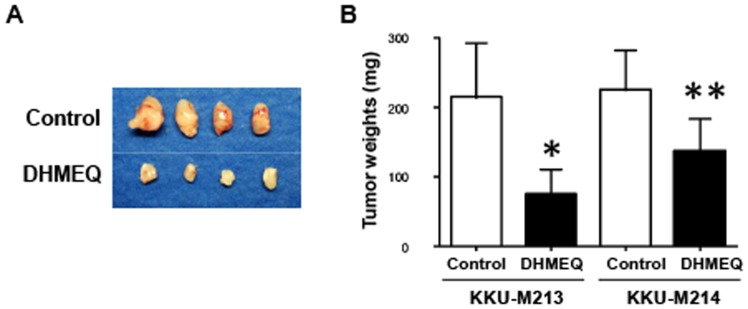
Inhibition of NF-κB action reduced growth of CCA in xenografted mice. Mice were subcutaneously injected with 4 × 10^6^ CCA cells at both flank sides. A day after tumor injection, mice were intraperitoneally injected with PBS (control) or DHMEQ at 10 mg/kg body weight daily for 17 days. A) CCA tumor tissues obtained from control and DHMEQ-treated mice. B) Comparison of tumor weights obtained from control and DHMEQ-treated mice. *P < 0.05, **P < 0.01; independent-sample t-test compared to the control group.

The NF-κB inhibitor was further examined as to whether DHMEQ suppressed growth of tumors in CCA-inoculated mice. CCA cell lines were subcutaneously injected in the flanks of NOJ mice, and a day after, DHMEQ was injected intraperitoneally daily for 17 days. Mice treated with DHMEQ were healthy and had similar body weights as the control mice injected with PBS. No side effects (body weight, physical activities and eating habits) were observed during DHMEQ treatment. Supplementation of DHMEQ significantly reduced tumor sizes (Fig. 5A) and tumor weights (Fig. 5B) in the mice inoculated with KKU-M213 (*P* < 0.05) and KKU-M214 (*P* < 0.001) as compared to the controls.

### Inhibition of NF-κB action induced cell death in CCA patient tissues

**Figure 6:**
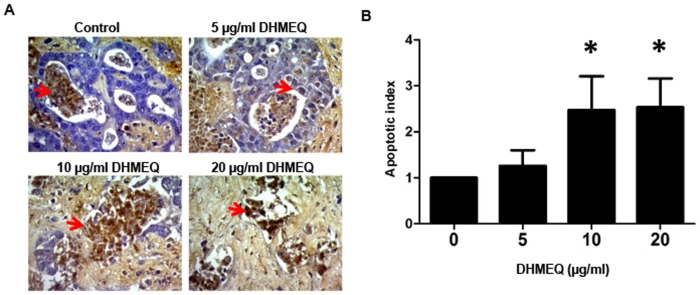
Inhibition of NF-κB action induced cell apoptosis in primary histoculture of CCA patient tissues. Tumor tissues of CCA patients (n  =  4) were cultured in medium containing 20% fetal calf serum in the absence or presence of 5, 10 and 20 µg/mL DHMEQ for 4 days. A) Apoptotic cells in tumor tissues were determined using the TUNEL assay. B) The number of apoptotic cells observed in DHMEQ-treated tissues was increased significantly compared with those of the control. Red arrows indicated TUNEL-positive nuclei (dead cells) and the blue stained cells were TUNEL-negative nuclei (alive cells), magnification ×40. *P< 0.05 independent-sample t-test compared to the control group.

The HDRA is the representative of an *in vitro* drug-response assay for anticancer agents [23], [24]. Several clinical studies have revealed that inhibition rates obtained from HDRA can predict the clinical responses to chemotherapy of corresponding patients. In the current study, HDRA of DHMEQ treatment were performed on tissue samples from 4 CCA patients and apoptotic cells were determined using TUNEL immunohistochemical staining. As shown in Fig. 6A, higher numbers of dead cells than the controls were obviously observed in tumor tissues cultured in the presence of DHMEQ in a dose dependent manner. As compared to the controls, the apoptotic indices of CCA tissues treated with 5, 10 and 20 µg/mL DHMEQ were 1.26 + 0.67, 2.47 + 1.58 and 2.53 + 1.25, respectively and were significantly higher than those of controls (*P* < 0.05, Fig. 6B).

## Discussion

A body of evidence indicates that regulations of aberrant NF-κB and the signaling pathways that control its activity are involved in cancer development and progression, as well as in drug resistance, especially during chemotherapy and radiotherapy [11]–[14]. NF-κB is activated or overexpressed in many cancers, both solid and hematopoietic malignancies. Therefore, inhibition of the NF-κB pathway indicates a strategy for treatment of several cancers. The present study reports for the first time that all CCA tissues overexpressed NF-κB and suppression of NF-κB action by a specific NF-κB inhibitor, DHMEQ significantly suppressed growth and induced apoptosis in CCA cell lines, in CCA inoculated mice and in primary histocultures of CCA patient tissues.

The NF-κB family of transcription factors is comprised of RelA (p65), RelB, c-Rel, NF-κB1/p50 and NF-κB2/p52. These proteins share a conserved Rel homology domain responsible for DNA binding, homodimerization or heterodimerization, nuclear localization function, and interaction with its regulatory inhibitors, IκB proteins. The present study demonstrated that NF-κB p50, p52 and p65, were aberrantly expressed in CCA patient tissues. The involvement of NF-κB in carcinogenesis of human CCA was observed in this study as normal bile duct epithelia and hepatocytes did not express NF-κB. In contrast, it was frequently expressed in precancerous hyperplastic and dysplastic biliary cells. This observation was confirmed in the liver fluke (*Opisthorchis viverrini*) induced CCA-hamster model [25]. Of note, all CCA tissues (48/48) and CCA cell lines examined had positive expressions of NF-κB. These data emphasize the significant roles of NF-κB in CCA development and raised the possibility of targeting NF-κB for therapy of CCA.

**Figure 7:**
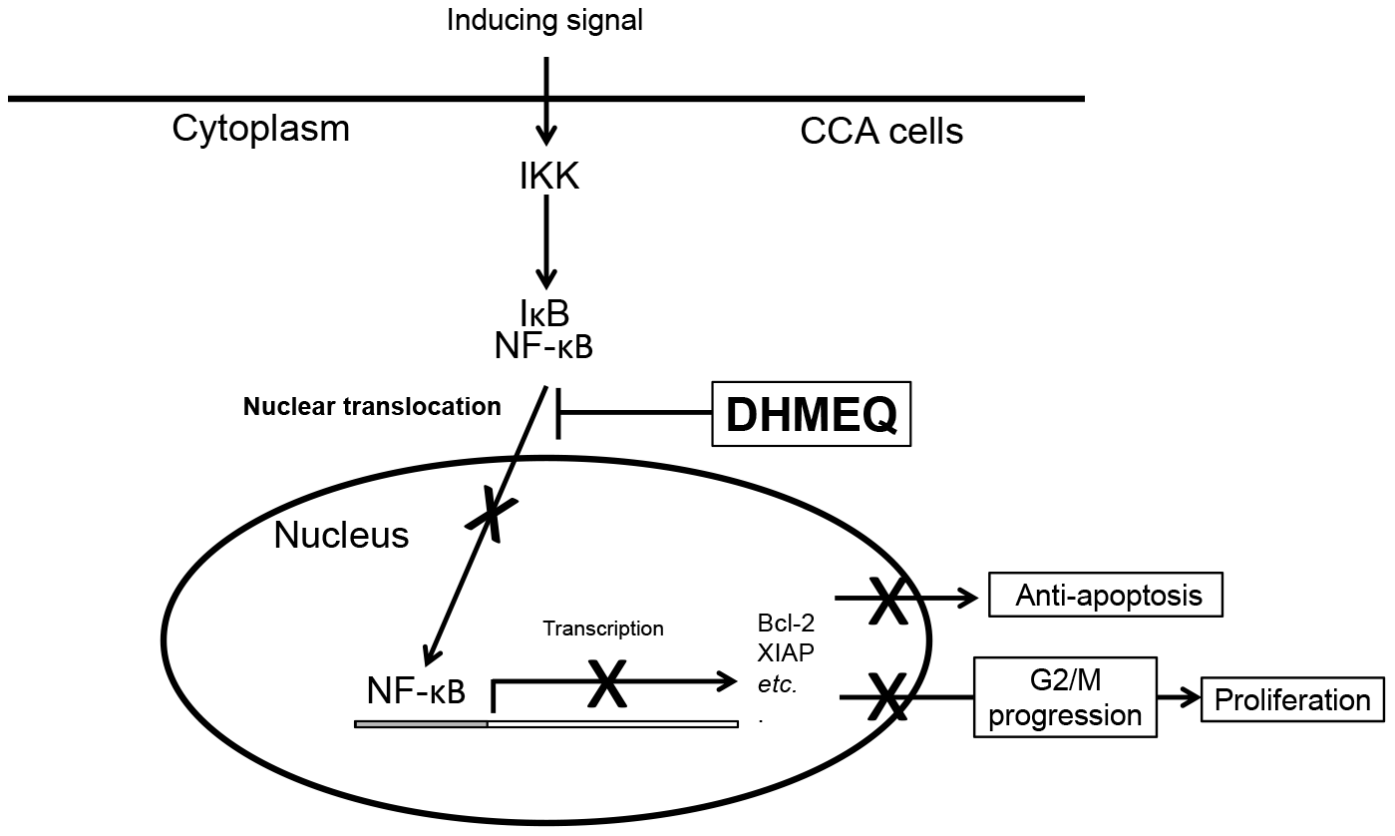
Molecular mechanisms of DHMEQ on NF-κB resulting in induction of cell cycle arrest and apoptosis in CCA cell lines are proposed.

Roles of NF-κB on growth and apoptosis have been demonstrated in several cancers. The effect of NF-κB signaling pathway on CCA cells, however, remains unclear. Thus, the purpose of the current study was to detect the role of the NF-κB signaling pathway on growth and apoptosis of CCA cells, *in vitro*, *in vivo* and *ex vivo*, using DHMEQ, an effective NF-κB inhibitor. DHMEQ can markedly inhibit the growth and induce apoptosis of CCA cell lines in a dose and time dependent fashions. Inhibition of NF-κB action by DHMEQ suppressed proliferation of five human CCA cell lines (KKU-M139, KKU-M156, KKU-M213, KKU-M214 and KKU-100) and induced cell cycle arrest at G2/M phase. Inhibiting nuclear translocation of NF-κB was shown to be the molecular action of DHMEQ [26]. DHMEQ could directly bind to the Rel-family proteins and inhibit their DNA binding activity [27]. However, as we could not find the NF-κB negative cell line to be used as a negative control for cytotoxicity test in this study, whether DHMEQ affects molecules other than NF-κB involved in cell death should be considered. The growth suppression effect of DHMEQ seems to correspond to the expression of NF-κB levels. KKU-100 with low expression of NF-κB (Fig. 1C) had lowest response to DHMEQ at all dose tested (Fig. 2A) comparing to other CCA cell lines with high expression of NF-κB. This indirectly signified the DHMEQ action on NF-κB in CCA cell lines. Similar effects of the inhibition of NF-κB on cell cycle arrest at the G2/M phase have been reported in lymphoma [28], [29], Non-Small-Cell Lung Cancer [30] and leukemia cells [31]. DHMEQ also induced cell apoptosis in CCA cell lines by suppression of Bcl-2 and XIAP, the anti-apoptotic proteins and activation of caspase-3 and caspase-9. These data suggest that DHMEQ induced apoptosis of CCA cells through the caspase pathway. The observation was confirmed in reports using other NF-κB inhibitors [32]–[34]. The molecular mechanism by which DHMEQ acts on induction of cell cycle arrest and apoptosis summarized from the current study is shown in Fig. 7.

Effects of DHMEQ on growth arrest and apoptosis of CCA cell lines were shown to be via inactivation of NF-κB. DHMEQ inhibited the nuclear translocation of all NF-κB subunits p50, p65 and p52 and suppressed the action of NF-κB. This action of DHMEQ is confirmed by many previous studies [35], [36]. The effectiveness of DHMEQ on growth arrest and apoptosis of CCA was demonstrated in CCA-inoculated mice and CCA patient tissue cultures. Mice treated with DHMEQ (10 mg/kg body weight) once a day for 17 days had significantly smaller tumor weights as compared to the controls, without side effects. Similar observations were reported in the animal models for bladder cancer [37], oral squamous cell carcinoma [38], thyroid cancer [35] and hepatoma [39].

DHMEQ is a novel NF-κB inhibitor derived from the structure of the antibiotic epoxyquinomicin. DHMEQ inhibits NF-κB transcriptional activity via blocking its translocation into the nucleus [40], [41]. The agent suppresses growth of various tumors, such as hormone-refractory prostate cancer, multiple myeloma, thyroid cancer cell, breast cancer, glioblastoma and squamous cell carcinoma *in vitro* and also in tumor-xenografted mice without any apparent side effects [38], [42]. Several chemicals and plant extracts were shown to suppress growth and induce apoptosis of CCA via inactivation of NF-κB, e.g., caffeic acid phenethyl ester [43], and curcumin [44].

The present authors have recently reported the efficacy of cepharanthine (CEP), a natural biscoclaurine alkaloid extract, on anti-proliferative activity [21], as well as inhibiting cell migration and invasion of human CCA cell lines [45]. CEP treatment effectively suppressed tumor growth in CCA-inoculated mice and also increased cell apoptosis in primary histocultures of CCA patient tissues. The molecular mechanism underlying CEP actions on cell growth and anti-metastasis was found to be through NF-κB inactivation. All these results emphasize the therapeutic potential of NF-κB inhibitors against human CCA.

Among the active compounds reported to suppress CCA growth via inactivation of NF-κB action, DHMEQ seems to be more potent than others. Treatment of DHMEQ at 10 mg/kg body weight for 17 days significantly suppressed tumor growth in mice whereas treatment of caffeic acid phenethyl ester at the same doses to the nude mice needed 77 days to show the similar effect [43].

The effect of DHMEQ on patient tissues determined by the histoculture drug response assay was first demonstrated in the present study. The apoptotic index as indicated by positive TUNEL staining was significantly higher in tissues cultured in the presence of DHMEQ in a dose-dependent manner. This three-dimensional, native state, histoculture assay simulates the tumor architecture in the body and may predict the responsiveness of tumors to DHMEQ if it is given to CCA patients. In addition to the growth suppression and induction of apoptosis, inhibition of NF-κB by DHMEQ also enhanced chemo- and/or radio-sensitivity of many cancer cells [46], [47].

In summary, this study demonstrated that all NF-κB subunits were over-expressed in CCA patient tissues. Inhibition of NF-κB action by DHMEQ induced cell cycle arrest and apoptosis in CCA cell lines, CCA inoculated mice and patient's histo-cultures. According to current available knowledge, this is the first study to report the possible involvement of NF-κB expression with growth and apoptosis of CCA cells. These findings suggest NF-κB as an attractive molecular target for CCA therapy. DHMEQ and other NF-κB inhibitors are the most promising anti-tumor compounds for treatment of CCA patients.

## References

[1] Sripa B, Pairojkul C (2008) Cholangiocarcinoma: lessons from Thailand. Curr Opin Gastroenterol 24: 349–356. 18408464 10.1097/MOG.0b013e3282fbf9b3 PMC4130346

[2] Sripa B, Brindley PJ, Mulvenna J, Laha T, Smout MJ, et al (2012) The tumorigenic liver fluke Opisthorchis viverrini–multiple pathways to cancer. Trends Parasitol 28: 395–407. 22947297 10.1016/j.pt.2012.07.006 PMC3682777

[3] Shin HR, Oh JK, Masuyer E, Curado MP, Bouvard V, et al (2010) Epidemiology of cholangiocarcinoma: an update focusing on risk factors. Cancer Sci 101: 579–585. 20085587 10.1111/j.1349-7006.2009.01458.x PMC11158235

[4] Srivatanakul P, Ohshima H, Khlat M, Parkin M, Sukaryodhin S, et al (1991) Opisthorchis viverrini infestation and endogenous nitrosamines as risk factors for cholangiocarcinoma in Thailand. Int J Cancer 48: 821–825. 1650329 10.1002/ijc.2910480606

[5] The Bureau of Policy and Strategy MoPh, Thailand (2010) Public health Statistic 2010.

[6] Kamsa-ard S, Wiangnon S, Suwanrungruang K, Promthet S, Khuntikeo N, et al (2011) Trends in liver cancer incidence between 1985 and 2009, Khon Kaen, Thailand: cholangiocarcinoma. Asian Pac J Cancer Prev 12: 2209–2213. 22296358

[7] Brown M, Cohen J, Arun P, Chen Z, Van Waes C (2008) NF-kappaB in carcinoma therapy and prevention. Expert Opin Ther Targets 12: 1109–1122. 18694378 10.1517/14728222.12.9.1109 PMC2605706

[8] Mauro C, Zazzeroni F, Papa S, Bubici C, Franzoso G (2009) The NF-kappaB transcription factor pathway as a therapeutic target in cancer: methods for detection of NF-kappaB activity. Methods Mol Biol 512: 169–207. 19347278 10.1007/978-1-60327-530-9_10

[9] Shen HM, Tergaonkar V (2009) NFkappaB signaling in carcinogenesis and as a potential molecular target for cancer therapy. Apoptosis 14: 348–363. 19212815 10.1007/s10495-009-0315-0

[10] Van Waes C (2007) Nuclear factor-kappaB in development, prevention, and therapy of cancer. Clin Cancer Res 13: 1076–1082. 17317814 10.1158/1078-0432.CCR-06-2221

[11] Fahy BN, Schlieman MG, Virudachalam S, Bold RJ (2004) Inhibition of AKT abrogates chemotherapy-induced NF-kappaB survival mechanisms: implications for therapy in pancreatic cancer. J Am Coll Surg 198: 591–599. 15051014 10.1016/j.jamcollsurg.2003.12.005

[12] Yeh PY, Chuang SE, Yeh KH, Song YC, Cheng AL (2003) Involvement of nuclear transcription factor-kappa B in low-dose doxorubicin-induced drug resistance of cervical carcinoma cells. Biochem Pharmacol 66: 25–33. 12818362 10.1016/s0006-2952(03)00250-8

[13] Cheng JC, Chou CH, Kuo ML, Hsieh CY (2006) Radiation-enhanced hepatocellular carcinoma cell invasion with MMP-9 expression through PI3K/Akt/NF-kappaB signal transduction pathway. Oncogene 25: 7009–7018. 16732316 10.1038/sj.onc.1209706

[14] Madhusoodhanan R, Natarajan M, Veeraraghavan J, Herman TS, Aravindan N (2009) NFkappaB activity and transcriptional responses in human breast adenocarcinoma cells after single and fractionated irradiation. Cancer Biol Ther 8: 765–773. 19276662 10.4161/cbt.8.9.8105

[15] Ban JO, Oh JH, Hwang BY, Moon DC, Jeong HS, et al (2009) Inflexinol inhibits colon cancer cell growth through inhibition of nuclear factor-kappaB activity via direct interaction with p50. Mol Cancer Ther 8: 1613–1624. 19509257 10.1158/1535-7163.MCT-08-0694

[16] Liu M, Ju X, Willmarth NE, Casimiro MC, Ojeifo J, et al (2009) Nuclear factor-kappaB enhances ErbB2-induced mammary tumorigenesis and neoangiogenesis in vivo. Am J Pathol 174: 1910–1920. 19349372 10.2353/ajpath.2009.080706 PMC2671278

[17] Furuta H, Osawa K, Shin M, Ishikawa A, Matsuo K, et al (2012) Selective inhibition of NF-kappaB suppresses bone invasion by oral squamous cell carcinoma in vivo. Int J Cancer 131: E625–635. 22262470 10.1002/ijc.27435

[18] Stein SJ, Baldwin AS (2011) NF-kappaB suppresses ROS levels in BCR-ABL(+) cells to prevent activation of JNK and cell death. Oncogene 30: 4557–4566. 21625221 10.1038/onc.2011.156 PMC3165082

[19] Godwin P, Baird AM, Heavey S, Barr MP, O'Byrne KJ, et al (2013) Targeting nuclear factor-kappa B to overcome resistance to chemotherapy. Front Oncol 3: 120. 23720710 10.3389/fonc.2013.00120 PMC3655421

[20] Solomon LA, Ali S, Banerjee S, Munkarah AR, Morris RT, et al (2008) Sensitization of ovarian cancer cells to cisplatin by genistein: the role of NF-kappaB. J Ovarian Res 1: 9. 19025644 10.1186/1757-2215-1-9 PMC2611983

[21] Seubwai W, Vaeteewoottacharn K, Hiyoshi M, Suzu S, Puapairoj A, et al (2010) Cepharanthine exerts antitumor activity on cholangiocarcinoma by inhibiting NF-kappaB. Cancer Sci 101: 1590–1595. 20412118 10.1111/j.1349-7006.2010.01572.x PMC11158067

[22] Sripa B, Leungwattanawanit S, Nitta T, Wongkham C, Bhudhisawasdi V, et al (2005) Establishment and characterization of an opisthorchiasis-associated cholangiocarcinoma cell line (KKU-100). World J Gastroenterol 11: 3392–3397. 15948244 10.3748/wjg.v11.i22.3392 PMC4315993

[23] Kodera Y, Ito S, Fujiwara M, Mochizuki Y, Ohashi N, et al (2006) In vitro chemosensitivity test to predict chemosensitivity for paclitaxel, using human gastric carcinoma tissues. Int J Clin Oncol 11: 449–453. 17180513 10.1007/s10147-006-0618-x

[24] Fujita Y, Hiramatsu M, Kawai M, Nishimura H, Miyamoto A, et al (2009) Histoculture drug response assay predicts the postoperative prognosis of patients with esophageal cancer. Oncol Rep 21: 499–505. 19148528

[25] Pinlaor S, Hiraku Y, Yongvanit P, Tada-Oikawa S, Ma N, et al (2006) iNOS-dependent DNA damage via NF-kappaB expression in hamsters infected with Opisthorchis viverrini and its suppression by the antihelminthic drug praziquantel. Int J Cancer 119: 1067–1072. 16570287 10.1002/ijc.21893

[26] Ariga A, Namekawa J, Matsumoto N, Inoue J, Umezawa K (2002) Inhibition of tumor necrosis factor-alpha -induced nuclear translocation and activation of NF-kappa B by dehydroxymethylepoxyquinomicin. J Biol Chem 277: 24625–24630. 11983688 10.1074/jbc.M112063200

[27] Yamamoto M, Horie R, Takeiri M, Kozawa I, Umezawa K (2008) Inactivation of NF-kappaB components by covalent binding of (−)-dehydroxymethylepoxyquinomicin to specific cysteine residues. J Med Chem 51: 5780–5788. 18729348 10.1021/jm8006245

[28] Hayashi S, Sakurai H, Hayashi A, Tanaka Y, Hatashita M, et al (2010) Inhibition of NF-kappaB by combination therapy with parthenolide and hyperthermia and kinetics of apoptosis induction and cell cycle arrest in human lung adenocarcinoma cells. Int J Mol Med 25: 81–87. 19956905

[29] Du HP, Shen JK, Yang M, Wang YQ, Yuan XQ, et al (2010) 4-Chlorobenzoyl berbamine induces apoptosis and G2/M cell cycle arrest through the PI3K/Akt and NF-kappaB signal pathway in lymphoma cells. Oncol Rep 23: 709–716. 20127010 10.3892/or_00000688

[30] Zhang L, Ruan J, Yan L, Li W, Wu Y, et al (2012) Xanthatin induces cell cycle arrest at G2/M checkpoint and apoptosis via disrupting NF-kappaB pathway in A549 non-small-cell lung cancer cells. Molecules 17: 3736–3750. 22450683 10.3390/molecules17043736 PMC6268665

[31] Uen YH, Liu DZ, Weng MS, Ho YS, Lin SY (2007) NF-kappaB pathway is involved in griseofulvin-induced G2/M arrest and apoptosis in HL-60 cells. J Cell Biochem 101: 1165–1175. 17226769 10.1002/jcb.21240

[32] Meng Z, Lou S, Tan J, Xu K, Jia Q, et al (2012) Nuclear factor-kappa B inhibition can enhance apoptosis of differentiated thyroid cancer cells induced by 131I. PLoS One 7: e33597. 22438958 10.1371/journal.pone.0033597 PMC3306418

[33] Hussain AR, Ahmed M, Al-Jomah NA, Khan AS, Manogaran P, et al (2008) Curcumin suppresses constitutive activation of nuclear factor-kappa B and requires functional Bax to induce apoptosis in Burkitt's lymphoma cell lines. Mol Cancer Ther 7: 3318–3329. 18852135 10.1158/1535-7163.MCT-08-0541

[34] Rahman KW, Sarkar FH (2005) Inhibition of nuclear translocation of nuclear factor-{kappa}B contributes to 3,3'-diindolylmethane-induced apoptosis in breast cancer cells. Cancer Res 65: 364–371. 15665315

[35] Starenki DV, Namba H, Saenko VA, Ohtsuru A, Maeda S, et al (2004) Induction of thyroid cancer cell apoptosis by a novel nuclear factor kappaB inhibitor, dehydroxymethylepoxyquinomicin. Clin Cancer Res 10: 6821–6829. 15501958 10.1158/1078-0432.CCR-04-0463

[36] Matsumoto G, Namekawa J, Muta M, Nakamura T, Bando H, et al (2005) Targeting of nuclear factor kappaB Pathways by dehydroxymethylepoxyquinomicin, a novel inhibitor of breast carcinomas: antitumor and antiangiogenic potential in vivo. Clin Cancer Res 11: 1287–1293. 15709200

[37] Kodaira K, Kikuchi E, Kosugi M, Horiguchi Y, Matsumoto K, et al (2010) Potent cytotoxic effect of a novel nuclear factor-kappaB inhibitor dehydroxymethylepoxyquinomicin on human bladder cancer cells producing various cytokines. Urology 75: 805–812. 20156648 10.1016/j.urology.2009.11.048

[38] Yasuda A, Kondo S, Nagumo T, Tsukamoto H, Mukudai Y, et al (2011) Anti-tumor activity of dehydroxymethylepoxyquinomicin against human oral squamous cell carcinoma cell lines in vitro and in vivo. Oral Oncol 47: 334–339. 21459660 10.1016/j.oraloncology.2011.03.001

[39] Nishimura D, Ishikawa H, Matsumoto K, Shibata H, Motoyoshi Y, et al (2006) DHMEQ, a novel NF-kappaB inhibitor, induces apoptosis and cell-cycle arrest in human hepatoma cells. Int J Oncol 29: 713–719. 16865289

[40] Umezawa K (2006) Inhibition of tumor growth by NF-kappaB inhibitors. Cancer Sci 97: 990–995. 16925581 10.1111/j.1349-7006.2006.00285.x PMC11158475

[41] Umezawa K, Chaicharoenpong C (2002) Molecular design and biological activities of NF-kappaB inhibitors. Mol Cells 14: 163–167. 12442886

[42] Fukushima T, Kawaguchi M, Yorita K, Tanaka H, Takeshima H, et al (2012) Antitumor effect of dehydroxymethylepoxyquinomicin, a small molecule inhibitor of nuclear factor-kappaB, on glioblastoma. Neuro Oncol 14: 19–28. 21968049 10.1093/neuonc/nor168 PMC3245990

[43] Onori P, DeMorrow S, Gaudio E, Franchitto A, Mancinelli R, et al (2009) Caffeic acid phenethyl ester decreases cholangiocarcinoma growth by inhibition of NF-kappaB and induction of apoptosis. Int J Cancer 125: 565–576. 19358267 10.1002/ijc.24271 PMC3051346

[44] Prakobwong S, Gupta SC, Kim JH, Sung B, Pinlaor P, et al (2011) Curcumin suppresses proliferation and induces apoptosis in human biliary cancer cells through modulation of multiple cell signaling pathways. Carcinogenesis 32: 1372–1380. 21325634 10.1093/carcin/bgr032 PMC3165121

[45] Uthaisar K, Seubwai W, Srikoon P, Vaeteewoottacharn K, Sawanyawisuth K, et al (2012) Cepharanthine suppresses metastatic potential of human cholangiocarcinoma cell lines. Asian Pac J Cancer Prev 13 Suppl: 149–154 23480757

[46] Kozakai N, Kikuchi E, Hasegawa M, Suzuki E, Ide H, et al (2012) Enhancement of radiosensitivity by a unique novel NF-kappaB inhibitor, DHMEQ, in prostate cancer. Br J Cancer 107: 652–657. 22805327 10.1038/bjc.2012.321 PMC3419964

[47] Suzuki K, Aiura K, Matsuda S, Itano O, Takeuchi O, et al (2013) Combined effect of dehydroxymethylepoxyquinomicin and gemcitabine in a mouse model of liver metastasis of pancreatic cancer. Clin Exp Metastasis 30: 381–392. 23111540 10.1007/s10585-012-9544-7

